# Sequence typing of *Haemophilus ducreyi* isolated from patients in the Namatanai region of Papua New Guinea: Infections by Class I and Class II strain types differ in ulcer duration and resurgence of infection after azithromycin treatment

**DOI:** 10.1371/journal.pntd.0012398

**Published:** 2024-08-15

**Authors:** Monica Medappa, Petra Pospíšilová, Lucy N. John, Camila González-Beiras, Marti Vall-Mayans, Oriol Mitjà, David Šmajs

**Affiliations:** 1 Department of Biology, Faculty of Medicine, Masaryk University, Brno, Czech Republic; 2 National Department of Health, Aopi Centre, Port Moresby, Papua New Guinea; 3 Skin Neglected Tropical Diseases and Sexually Transmitted Infection section, Fight Infectious Diseases Foundation, University Hospital Germans Trias i Pujol, Badalona, Spain; 4 Universitat Autónoma de Barcelona, Bellaterra (Cerdanyola del Vallés), Spain; Yale University School of Medicine, UNITED STATES OF AMERICA

## Abstract

*Haemophilus ducreyi* (HD) is an important cause of cutaneous ulcers in several endemic regions, including the Western Pacific Region, especially among children. An HD sequence typing on swab samples taken from 1,081 ulcers in the Namatanai district of Papua New Guinea, during the pilot study for treatment of yaws, has been performed using the Grant typing system. Of the 363 samples that tested positive for the 16S rDNA of HD, the *dsrA* sequences of 270 samples were determined. Altogether they revealed 8 HD strain types circulating in Namatanai, including seven strain types of Class I (I.3, I.4, I.5, I.9, I.10, I.11, I.12) and one strain of Class II (II.3); four Class I types (I.9, I.10, I.11, I.12) were novel. The southern region of Namatanai (Matalai Rural) was identified as the region with the lowest genotype diversity and with most infections caused by HD Class II. The middle and northern subdistricts were affected mainly by HD Class I. Analysis of patient characteristics revealed that Class II HD infections were more often represented by longer-lasting ulcers than Class I HD infections. An increase in the prevalence of the I.10 strain was found after azithromycin administration compared to the untreated population at baseline likely reflecting higher infectivity of HD Class I, and more specifically strain type I.10.

## Author summary

*Haemophilus ducreyi* (HD) is one of the main causative agents of cutaneous ulcers in children across substantial parts of Africa and the Pacific. A yaws eradication study conducted in the Namatanai district of Papua New Guinea revealed that nearly one-third of all patients were infected with HD. Single locus sequence typing (SLST) of the HD *dsrA* locus revealed presence of multiple HD strains circulating in the human population of Namatanai region. In contrast to *Treponema pallidum* subsp. *pertenue*, the pathogen responsible for yaws, the prevalence of HD strains did not significantly decrease following mass drug administration. In addition, HD strains from patients in Namatanai region revealed a higher genetic diversity compared to the yaws agent present in the same area and the HD strain composition changed following antibiotic treatment. Infections caused by at least some different HD strain types appear to differ in reported ulcer duration and also in resurgence of infection after antibiotic treatment. Therefore, important pathophysiological differences among HD strain types were predicted.

## Introduction

*Haemophilus ducreyi* (HD) is an important cause of cutaneous ulcers in the endemic regions of Africa, Polynesia, Micronesia, and Melanesia, especially among children [1]. HD also causes sexually transmitted genital ulcers (chancroid) in adults, and these ulcers are more common in men compared to women [2]. There are two classes of HD, Class I and Class II, based on the genetic diversity in several pathogenicity-related genes. Both classes were shown to cause genital and cutaneous ulcers, and no differences in clinical outcomes between classes have been observed to date [3].

Since ulcers caused by HD are morphologically indistinguishable from yaws ulcers caused by *Treponema pallidum* (TP) subsp. *pertenue* (TPE), we analyzed samples taken within a pilot TPE eradication study in Papua New Guinea for the presence of HD DNA. Then, we typed the individual samples of HD [4]. A study by John et al. [4] analyzed swab samples taken from 1,081 ulcers from trial participants in the Namatanai district of Papua New Guinea, however, due to lack of clinical and demographic data, 78 samples were subsequently excluded leaving a total of 1,026 analyzed skin ulcers. Of 1,081 samples, 302 were confirmed to be yaws ulcers based on PCR amplification of TPE *polA* gene.

Based on previously published studies [5–7] that identified skin ulcerations resembling yaws lesions caused by HD, we hypothesized that we could detect HD positivity in ulcer swabs collected for yaws study. The study by Grant et al. [7] introduced a single locus HD typing system based on *dsrA* sequencing and revealed that multiple HD strains circulated in the infected human population living on Lihir Island, Papua New Guinea (PNG). Moreover, the HD strain composition in the infected population was unaffected by antibiotic pressure following one round of total community treatment (TCT) despite the fact that a single oral dose of azithromycin has been successfully tested for treatment of ulcers caused by HD [4,8,9]. Similarly, the study by John et al. [4] revealed that three rounds of TCT in Namatanai (New Ireland Province, PNG) were equally effective in decreasing the prevalence of HD as one round of TCT followed by total targeted treatments.

In this study, we performed HD sequence typing on DNA isolated from swab samples of 363 HD-positive ulcers isolated from patients in the Namatanai region of Papua New Guinea for treatment of yaws [4].

## Material and methods

### Ethics statement

All the trial participants (for children, their parents, or guardians) provided verbal and written informed consent for screening and treatment. The study protocol was approved by the Medical Research Advisory Committee of the Papua New Guinea National Department of Health (MRAC No: 17.19), which authorized oral consent [4].

### Collection of samples

Samples originated from participants living in the Namatanai district of Papua New Guinea who took part in a previously reported study that described a 1.5-year yaws eradication campaign held in Namatanai between June 2018 and December 2019 [4,10]. Swab samples were collected from patients with exudative ulcers on the lower limbs, placed in cryotubes with 1 ml of lysis buffer (100 mM TRIS, pH = 8; 100 mM EDTA, pH = 8; 1% SDS) and stored in a freezer at -20°C. They were subsequently transported in a lysis buffer from Papua New Guinea to the Czech Republic [4], where they were further analyzed.

### Analysis of samples

DNA was purified using QIAquick Purification Kit (QIAGEN Valencia, CA, USA). All 1,081 samples were tested for HD PCR positivity targeting the 16S rRNA gene using qPCR. Initial screen for HD DNA in the samples was performed by qPCR detection. Primers and probe targeting 16S rDNA were adapted from Orle et al. [11]. The TaqMan probe had the following sequence: 5’–FAM-CCGAAGGTCCCACCCTTTAATCCGA-BHQ–3’. Primer sequences were as follows: 5’–CAAGTCGAACGGTAGCACGAAG–3’ and 5’–TTCTGTGACTAACGTCAATCAATTTTG–3’. The qPCR was performed using the QuantStudio 3 Real Time PCR System (Thermo Fisher Scientific). Each PCR reaction (20 μl) contained Luna Universal Probe qPCR Master Mix (10 μl, New England Biolabs), primers (0.08 μl each, final conc. 400 nM), FAM-labeled probe (0.04 μl, final conc. 200 nM), template DNA (1μl), and nuclease-free water (8.8 μl). PCR cycling conditions were 95 ⁰C (3 min), followed by 40 cycles at 95 ⁰C (10 s), and 60 ⁰C (30 s). All samples with Ct values lower than 34 were considered positive.

For molecular typing, *dsrA* locus amplification was performed for all 16S rDNA-positive samples. Typeable samples (n = 270) were considered those where the *dsrA* locus of HD was successfully amplified and sequenced. Primers for the *dsrA* locus were taken from Grant et al. [7] and targeted the *dsrA* locus of HD Class I (*dsrA*1) and HD Class II (*dsrA*2). Sequencing of *dsrA* locus was used to classify HD DNAinto strain types, using the same numbering system as described previously [7]. The *dsrA*1 primers targeted chromosomal regions between base pairs 603,481–603,921 of the HD reference strain 35000HP (GenBank acc. no. AE017143.1). whereas the *dsrA*2 primers targeted regions between base pairs 555,370–556,080 of the HD reference strain CIP54.2 (GenBank acc. no. CP011229.1). The *dsrA*1 primers did not amplify strains belonging to strain type I.7 that was described by Pillay et al. [12]. A modified forward primer *dsrA*1v2, which was four nucleotides shorter than the *dsrA*1 forward primer at the 3’ end was employed to amplify I.7 strain types because these strain types had a three nucleotide (AAT) deletion in the corresponding DNA sequence. The primer sequences are shown in Table 1. To test potential macrolide resistance mutations, the 23S rRNA gene locus was amplified and analyzed between coordinates 1595–2279 of the 23S rRNA genes using primers listed in Table 1.

**Table 1:** Primer sequences targeting the *dsrA* locus of HD Class I and Class II and 23S rDNA of HD Class I.

**Primer sequences targeting the *dsrA* locus of HD Class I and Class II**		
Class/strain type	*dsrA*1 forward primer	*dsrA*1 reverse primer
**Class I: all types except I.7**	5’-AGGGTAAATGGA-CTTGGTCTAATG-3’	5’-TGGCTAAACCAGTTT-GCAATTC-3’
	*dsrA*1v2 forward primer	*dsrA*1 reverse primer
**Class I: I.7**	5’-AGGGTAAATGGA-CTTGGTCT-3’	5’-TGGCTAAACCAGTTT-GCAATTC-3’
	*dsrA*2 forward primer	*dsrA*2 reverse primer
**Class II**	5’-GGCATCAAACGGCTC-TTTATC-3’	5’-GCTAACGCACTCTTA-CCTCTAT-3’
**Primer sequences targeting 23S rDNA of HD Class I**		
Class/strain type	23S rRNA gene forward primer	23S rRNA gene reverse primer
**Class I: 35000HP**	5’-GTACTATAAACCGACACAGGT-3’	5’-CCTCCGTTACTCTTTGG-3’

The sizes of the PCR products without the primer sequences are as follows: Class I: *dsrA*1 forward primer-*dsrA*1 reverse primer (AE017143.1; 603505 to 603899; 395 bp), strain types I.7 of Class I: *dsrA*1v2 forward primer-*dsrA*1 reverse primer (CP015431.1 and CP015432.1; 247064 to 247480 and 343601 to 344017 respectively, 417 bp), and Class II: *dsrA*2 forward primer-*dsrA*2 reverse primer (CP015432.1; 555391 to 556058, 668 bp). The size of the 23S rDNA PCR product is 679 bp (641 bp without the primer sequences).

PCR amplification was performed using PrimeSTAR GXL polymerase (Takara Bio Inc., Otsu, Japan), at a total volume of 25 μl consisting of 5× PrimeStar GXL buffer (5 μl), 100μM primer stock solution (0.095 μl), PrimeSTAR dNTP mix (2 μl), and PrimeSTAR GXL DNA polymerase (0.5 μl), and the tested DNA (1 μl), under the following cycle conditions: 94°C initial denaturation for 1 minute, 8 cycles at 98°C (10 s), 68°C (15 s) with touch down protocol of -1°C per cycle and 68°C (1 minute 45 s), followed by 35 cycles at 98°C (10 s), 61°C (15 s), and 68°C (1 minute 45 s), with the final extension done at 68°C (7 minutes).

PCR products were Sanger sequenced bidirectionally by Eurofins Genomics (Constance, Germany). The obtained chromatogram files (.ab1 files) were trimmed on either side to eliminate primer sequences and bad-quality sequences. The data was aligned with the *dsrA* locus of HD 35000HP and HD CIP54.2 for Class I positive and Class II positive samples, respectively. The sequencing reads were edited using the Lasergene’s Editseq sequence editor program (DNASTAR Lasergene, EditSeq v.7.1.0; DNASTAR, Madison, WI, USA); consensus sequences were generated using the Seqman sequence assembling program (Lasergene, DNASTAR v.7.1.0; DNASTAR, Madison, WI, USA).

### Patient characteristics

Metadata was available for 346 HD-infected patients. Patients for whom metadata was unavailable were excluded from the analyses (n = 17). The study participants were divided into two arms: the control arm, which received one round of mass-drug administration (MDA), and the experimental arm, which received three rounds of MDA. The dataset comprised 30 distinct features (both categoric and numeric). We considered only the variables relevant to the study design, including age, sex, study rounds (R1, R2, R3, and R4), local-level government areas (Sentral Niu Ailan Rural, Namatanai Rural, and Matalai Rural), ulcer duration in weeks, number of ulcers, size of ulcer(s), treponemal-specific and -nonspecific serology (T-line, NT-line, T-reader, and NT-reader) [13], TPE positivity, and HD positivity. The T-line and NT-line refer to specific lines on the Chembio DPP Syphilis Screen & Confirm kit (https://chembio.com) that detect antibodies against TP and non-TP antigens in blood, respectively. The T-reader and NT-reader are used to quantitatively measure the optical density of these lines using microreaders [13].

### Statistical analysis

Demographic data, clinical characteristics, serological data and prevalence of HD strain types at baseline and post MDA were tested for possible associations using Pearson’s chi-square test and IBM SPSS Version 29.0.0.0. Variables with numerical values were converted to categorical values. The goodness of fit model was used to compare observed and expected frequencies. A p ≤ 0.05 was considered statistically significant.

In our analysis, the average Ct values of individual samples were calculated for 16S rRNA gene amplifications. Altogether, fourteen 96-well qPCR plates with patients’ samples were evaluated at various time points. Normalisation among plates was performed by using the swab sample T19.ba.2 that served as the control sample in 9 tested plates (labelled as 12a, 12b, 12B+0.98, 13a, 13b, 6a, 11a, 11b and 2019_12_04). The remaining 5 plates were excluded from the analysis as a different control sample (T19.ba.1) was used. Plate 11b was chosen as the reference and the samples from the remaining plates were calibrated against T19.ba.2 reference to plate 11b. In our analysis, data was normalised for 104 samples out of 270 HD-typed samples and the average Ct values were calculated for both normalised (n = 104) and non-normalised data sets (n = 270). Variables were tested for normal distribution with the Kolmogorov-Smirnov test and analyzed using a one-sample *t*-test. The level of significance was set to p ≤ 0.05.

### Phylogenetic analysis of HD strain types

The maximum likelihood method, employing the Tamura-Nei model, was utilized to construct the phylogenetic tree of HD strain types that were identified in this and previous studies [7].

## Results

### Characteristics of the study participants

During the yaws eradication study performed in Namatanai (District of New Ireland Province of Papua New Guinea), between June 2018 and December 2019 [4], a total of 1,081 swab samples were analyzed for the presence of HD DNA. The relevant characteristics of the study participants (n = 1,002), of HD-positive patients and of patients with typed HD samples are shown in Table 2. Most participants were below 15 years of age, with single or multiple ulcers that, in most cases, lasted up to three months.

**Table 2:** General characteristics of the study participants.

Characteristics of study participants (n = 1002)	No. of study participants (n = 1002*)	HD positive patients** (excluding coinfections with TPE) (n = 310*)	Typed HD isolates*** (excluding dual infections with II.3) (n = 245*)
Sex (M/F)	523/459* (n = 982)	123/141 (n = 264)	111/118 (n = 229)
Age (up to 9y/10-15y/more than 15y)	364/313/298 (n = 975)	106/83/70 (n = 259)	113/66/50 (n = 229)
No. of ulcers (1-2/more than 2)	812/105 (n = 917)	210/31 (n = 241)	192/20 (n = 212)
Diameter of ulcers (less than 2 cm/2 cm and more)	343/539 (n = 882)	103/133 (n = 236)	110/100 (n = 210)
Duration of ulcers (0–3 months/more than 3 up to 6 months/more than 6 months)	796/64/45 (n = 905)	213/11/7 (n = 231)	186/13/7 (n = 206)
Ulcer episode (first/multiple)	752/206 (n = 958)	199/59 (n = 258)	169/54 (n = 223)

* The patient characteristics were unavailable for some samples.

** 16S rDNA positive samples

*** *dsrA* typed samples

### PCR detection of HDin ulcer swab samples

Swab samples (n = 1,081) from lower limb ulcerations were primary collected to detect the yaws agent (TPE). However, these samples were also analyzed for HD. HD 16S rDNA was detected using realtime PCR in about a third of the samples (33.6%, n = 363) collected from the study participants. Of the 363 study samples with detectable amounts of HD DNA, *dsrA* gene sequences of 270 samples were determined; 93 samples were excluded due to PCR-negative *dsrA* gene amplification or ambiguous or incomplete DNA sequences. The relevant characteristics of the HD positive patients as well as patients with typed HD are shown in Table 3.

**Table 3:** Detected HD strain types, the number of patient samples with the same strain type, and the corresponding Ct values. A total of 363 (33.6%) samples were positive for the presence of HD 16S rDNA. In addition to detection of HD positivity, the average Ct values were calculated for HD strain types and ranged from 13 to 33.71.

Detected strain type	Sequence identity to previously characterized strain/novel sequence type	No. of identified *dsrA* sequences	No. of samples	Average Ct value of 16S rDNA detection (SD)* for normalized values	Average Ct value of 16S rDNA detection (SD)** for non-normalized values
I.3	AUSPNG1	53	53	27.5 (3.5) (n = 24)	26.6 (3.6) (n = 53)
I.4	NZS4, NZV1, VAN1, VAN3, VAN4, VAN5	15	15	N/A	26.1 (3.6) (n = 15)
I.5	NZS2 and NZS3	20	20	28.8 (3.1) (n = 12)	27.5 (3.0) (n = 20)
I.9	novel type (CZPNG1)	52	52	27.9 (3.4) (n = 16)	26.0 (3.5) (n = 52)
I.10	novel type (CZPNG2)	41	41	24.4*** (2.4) (n = 16)	23.3*** (2.8) (n = 41)
I.11	novel type (CZPNG3)	1	1	N/A	N/A
I.12	novel type (CZPNG4)	1	1	N/A	N/A
II.3	VAN2	112	87	26.9 (4.0) (n = 36)	25.4 (3.7) (n = 112)

* Only samples containing single *dsrA* sequence were analyzed excluding those co-infected with Class I and II HD strains

**Samples including those co-infected with Class I and II HD strains were analyzed

***p ≤ 0.001 when compared to Ct values obtained for other strain types; N/A, not applicable; SD, standard deviation of the mean

### Molecular typing of HD

**Fig 1:**
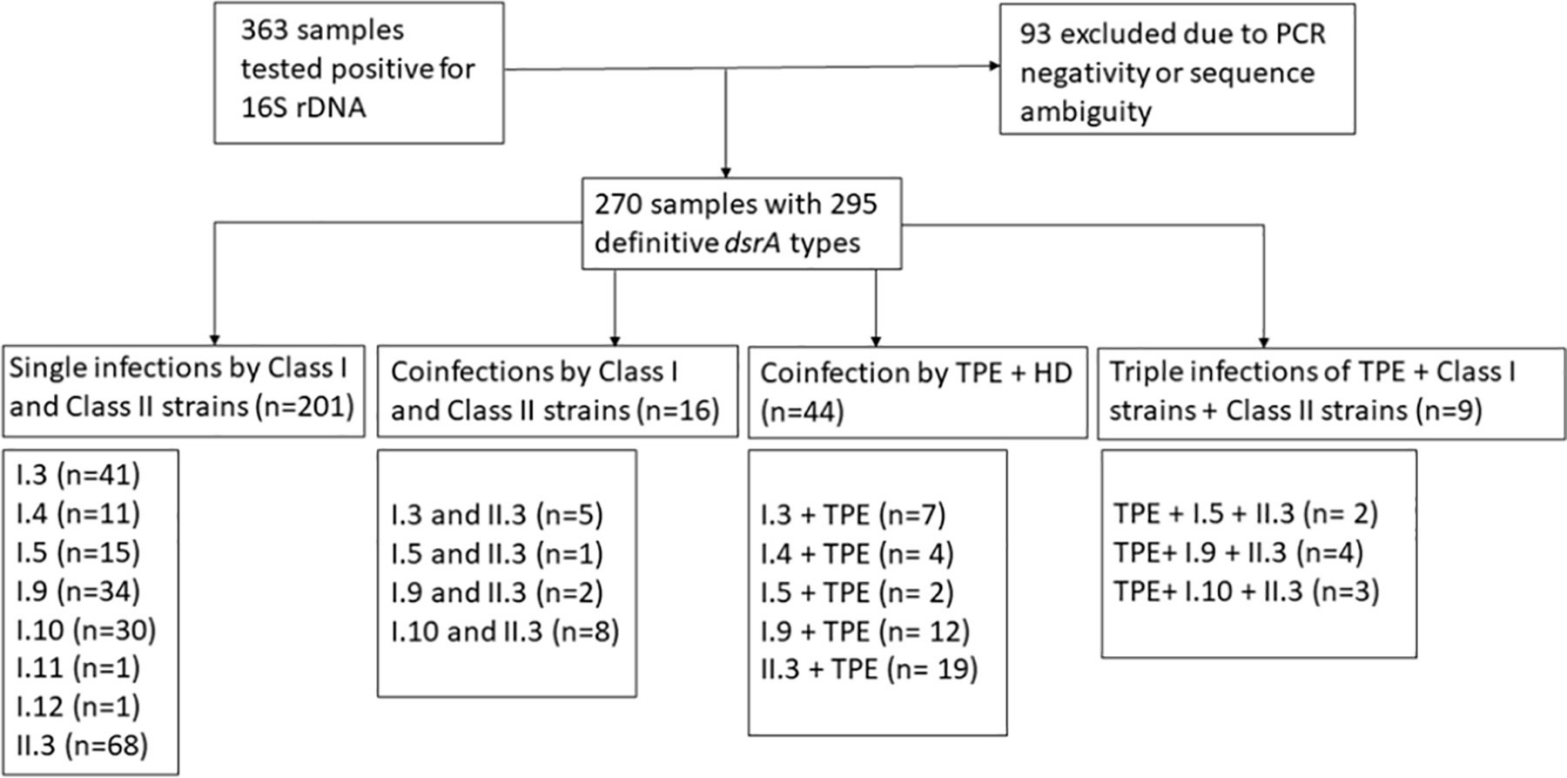
Overview of samples testing positive for HD DNA and identified HD strain types. A total of 363 (33.6%) samples were positive for the presence of HD 16S rDNA. The overview of samples analyzed in this study is shown. Most samples contained only a single *dsrA* sequence (n = 201) or *dsrA* sequences with TPE DNA (n = 44); a minority of samples contained multiple *dsrA* sequences (i.e., Class I and Class II sequence types, n = 25), which resulted in a higher number of determined sequences than the number of analyzed samples. Of the 93 excluded samples, 77.4% (n = 72) were PCR negative for *dsrA* detection and 23.6% (n = 21) generated ambiguous sequences during PCR amplicon sequencing. Of the 363 HD positive samples, a total of 72 samples (19.8%) were 16S rDNA positive but *dsrA* negative.

The *dsrA* gene sequencing (270 samples) revealed 295 individual *dsrA* sequences (the number of individual *dsrA* sequences is higher due to presence of two sequence types in some samples) and 8 HD strain types including 7 strain types of Class I (i.e., three types, I.3, I.4, I.5, previously described by Grant et al. [7] and additional types identified here: I.9, I.10, I.11, I.12) and one strain type of Class II (II.3) previously identified by Grant et al. [7]. The nomenclature adhered to the Grant typing system [7], which identified several HD strain types in population of Lihir Island. Consequently, the four new HD Class I strains identified in this study, namely I.9, I.10, I.11 and I.12, were classified in a similar manner to maintain continuity with the previous findings. A detailed scheme of results obtained by *dsrA* gene sequencing is shown in Fig 1. The detected strain types and the corresponding frequencies of these types are shown in Table 3. Of 270 samples, a total of 44 (16.3%) samples contained the DNA of both TPE and HD (Fig 1). In 25 cases, double HD coinfections were identified: II.3 and I.3 (n = 5), II.3 and I.5 (n = 3), and II.3 and I.9 (n = 6), and II.3 and I.10 (n = 11) (Fig 1).

**Fig 2:**
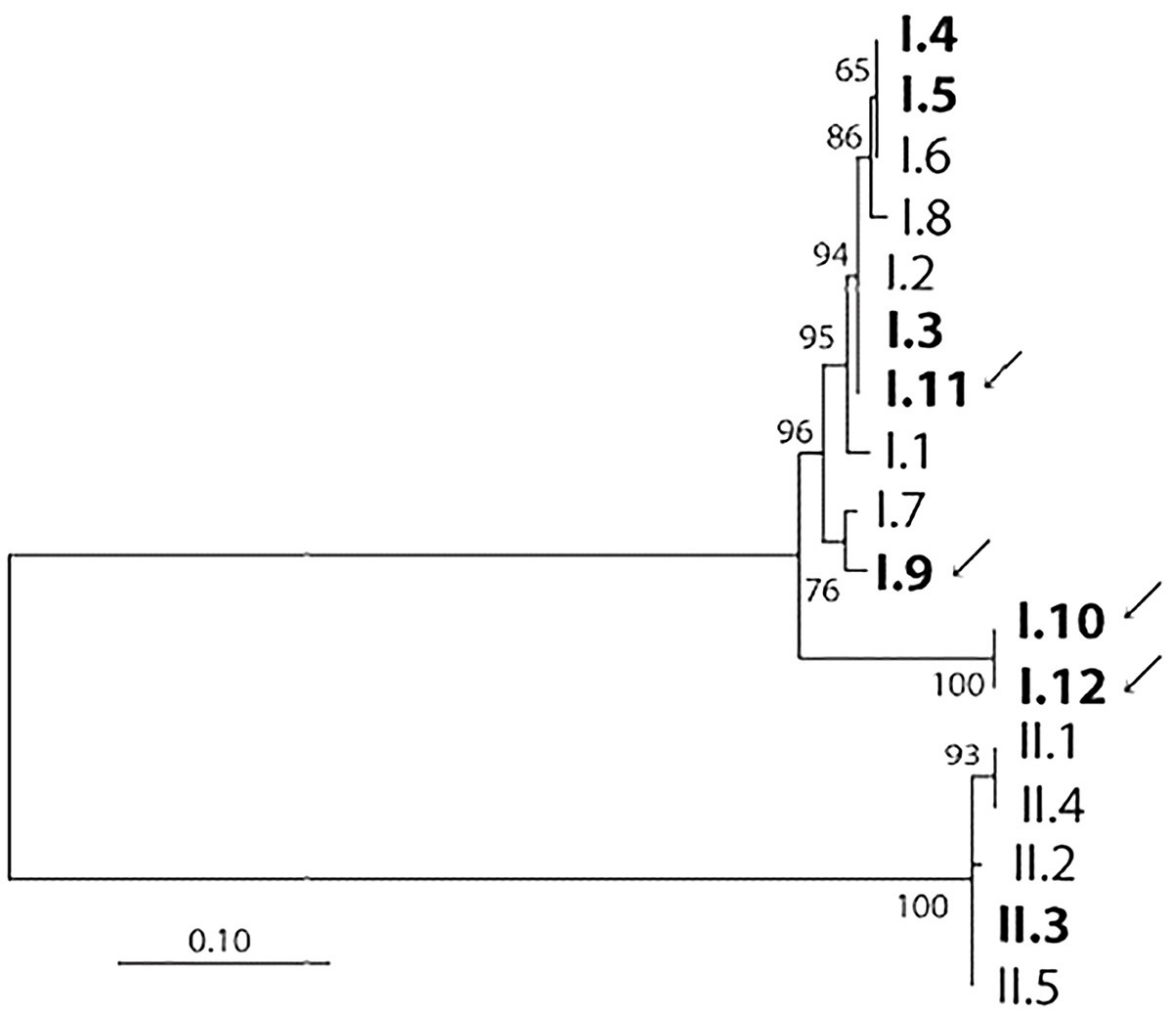
A phylogenetic tree of identified and novel strain types of HD. HD strain types identified in this study are shown in bold, and novel genotypes of Class I (denoted as I.9, I.10, I.11, and I.12) based on the typing of the *dsrA* gene [7] are indicated by arrows. The evolutionary history was inferred using the Maximum Likelihood method based on the Tamura-Nei model [14]. The percentage of trees in which the taxa clustered is shown next to the branches. The tree is drawn to scale, with branch lengths measured in the number of substitutions per site. The analysis involved 17 nucleotide sequences. There were 291 positions of the *dsrA* gene in the final dataset.

Four HD strain types of Class I were identified as novel sequences and were denoted as I.9, I.10, I.11, and I.12 (GenBank accession numbers PP266021—PP266024). The corresponding sequences of the *dsrA* gene were compared to existing sequences, and the phylogenic relatedness of the novel strains to previously described types is shown in Fig 2.

### Geographical localization of the identified HD genotypes in Namatanai, PNG

**Fig 3:**
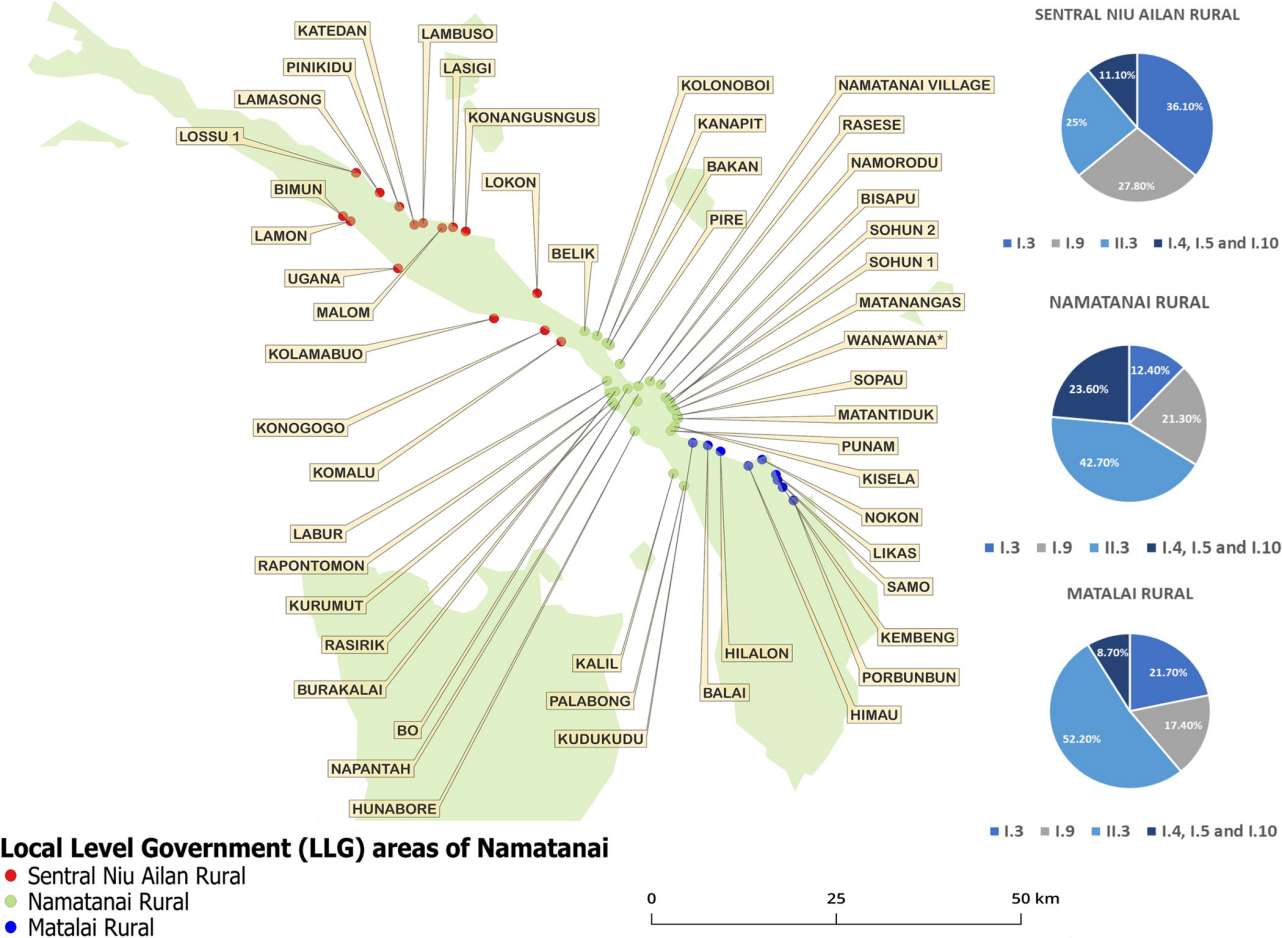
HD strain types found in three local-level government areas of Namatanai, Papua New Guinea, and the number of detected cases. Geographical data was only available for participants who were randomised at Round 1 (baseline) from both arms, with no data available for participants in subsequent rounds (Rounds 2, 3 and 4). The Round 1 contained a total of 133 participants which were included in the analysis. Data for 133 patients from baseline revealed the presence of six genotypes, including 15 coinfections, i.e., II.3 (n = 59), I.9 (n = 33), I.3 (n = 29), I.4 (n = 10), I.5 (n = 5), and I.10 (n = 12). This map was constructed using QGIS 3.30.2’ s-Hertogenbosch; the base layer made with Natural Earth. Free vector and raster map data @ naturalearthdata.com.

HD strain types detected in three local-level government areas of Namatanai, covering an area of over 2400 km^2^, are shown in Fig 3. Among the 29 villages in the southern part of Namatanai (MAT region), 21 cases of HD infections were found in a total of 9 villages. Similarly, among the 51 villages in central Namatanai (NTI region), there were 79 cases of HD infections in 28 villages Lastly, among the 73 villages of the northern part of Namatanai (SNA region), we detected 33 cases of HD infections in 16 villages. While there were more Class I HD strains, especially I.3, in the northern part of the Namatanai (SNA region), the southern part (MAT region) exhibited a higher frequency of Class II HD strains (II.3). Village information was available for all participants of the study at baseline [4] (Fig 3).

### Genotypes of HD identified in Namatanai before and after azithromycin treatment

**Fig 4:**
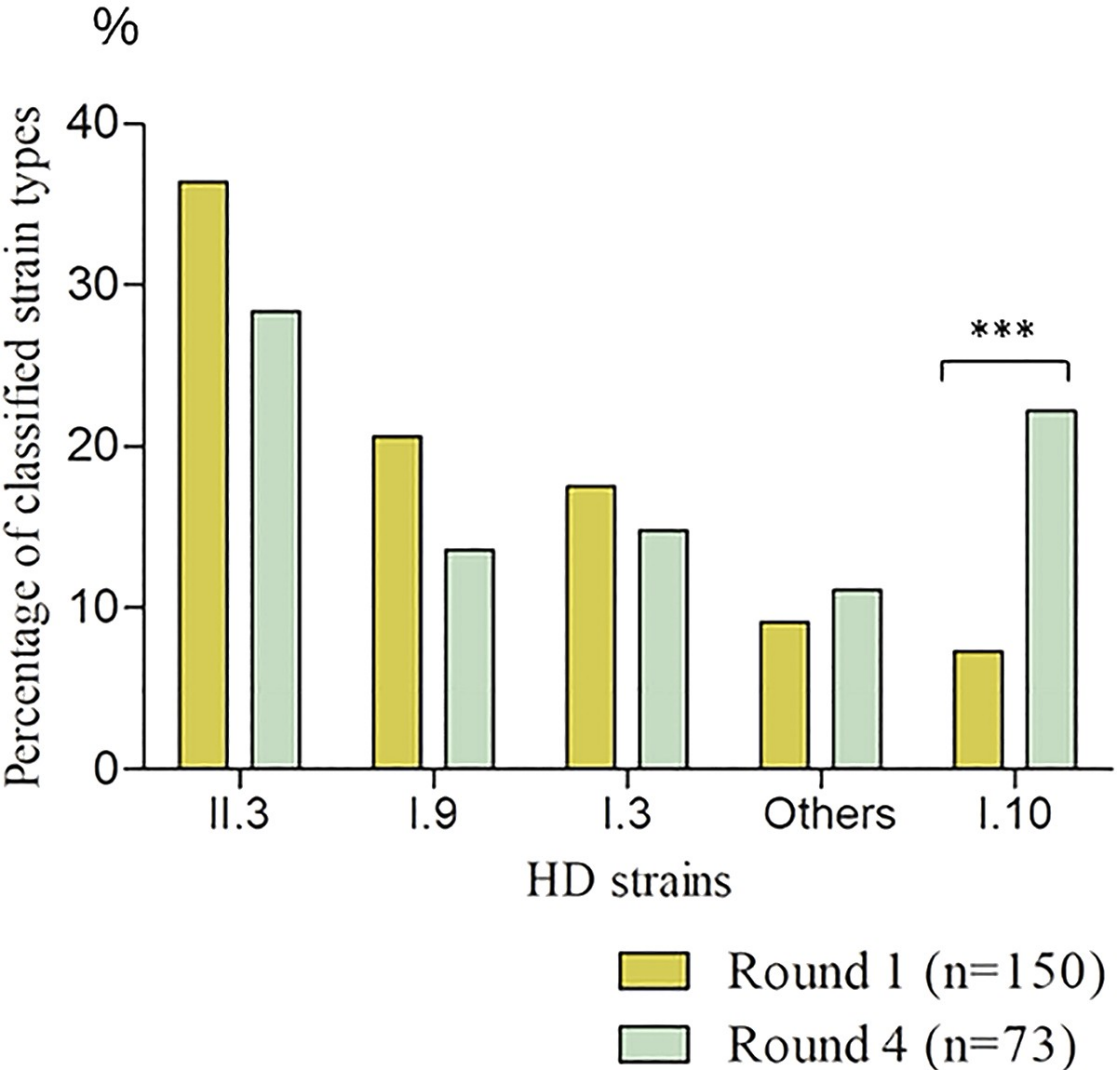
HD genotypes detected prior to and post-antibiotic treatment. HD genotypes were detected in Namatanai, Papua New Guinea, during Round 1 (n = 150) and Round 4 (n = 73) of the (previously mentioned) yaws eradication study [4], altogether, results from 223 typed samples from Round 1 and Round 4 are shown. In Fig 4, “Other” refers to minor genotypes such as I.4 (n = 14), I.5 (n = 9) and I.12 (n = 1). The prevalence of I.10 in the control arm increased significantly from 5.8% at baseline (Round 1) to 33.3% at Round 4 after MDA (p = 0.0008) while in the experimental arm, the prevalence of I.10 remained constant at 9% at baseline and 9.75% after MDA. A statistically significant increase in the percentage of the I.10 strain during Round 4 was detected (p = 0.0001). This graph was constructed using GraphPad PRISM, version 5.00.

Fig 4 shows the HD genotypes detected prior to (Round 1) and post-antibiotic treatment (Round 4) in Namatanai, Papua New Guinea. Interestingly, a notable increase in I.10 strain type prevalence was detected during the 18^th^ month (Round 4) of the study in both arms (p = 0.0001). Additionally, a novel I.12 strain type, resembling I.10 was detected in a single sample.

### Analysis of 23S rRNA genes with respect to potential mutations causing azithromycin resistance among the I.10 strains at baseline and at 18^th^ month of study (Round 4)

The increased prevalence of I.10 strains in Round 4 prompted us to detect for point mutations in 23S rRNA genes in I.10 samples at baseline (n = 12) and post-mass drug administration (MDA) (n = 18). The region of the I.10 strains spanning nucleotide positions between 1595–2279 of 23S rRNA genes of Class I strains were amplified and compared to the sequence of the Class I strain 35000HP (GenBank acc. no. AE017143.1). Since there are no previous reports of macrolide resistance-encoding mutations in HD, nucleotides in positions where macrolide-resistant point mutations had been found in *Haemophilus influenzae* were analyzed [15]. While most analyzed sequences were identical to Class I strain 35000HP (GenBank acc. no. AE017143.1), two distinct point mutations were identified in post-MDA in two I.10 samples: G1649A and C2092T. Futhermore, 23S rRNA genes were tested among control samples from Round 4 including I.3, I.4, I.5, and I.9 strain types. The sequences of I.5 and I.4 strain types revealed a C1754T substitution (Table 4). The association of these mutations with resistance is currently unknown.

**Table 4:** Analysis of 23S rRNA genes in HD I.10 and control strains from Round 1 and 4.

Strain type	Round in the pilot study for the treatment of yaws	Sequence analysis, compared to HD 35000HP	No. of analyzed samples	Total no. of samples in the round
I.10	R1	identical	n = 10	n = 12
I.10	R4	identical	n = 12	n = 18
		G1649A, C2092T	n = 2	
I.3	R4	identical	n = 1	n = 32
I.4	R4	C1754T	n = 1	
I.5	R4	C1754T	n = 3	
I.9	R4	identical	n = 3	
II.3	R4	N/A	not tested	n = 23

N/A, not applicable.

### Characteristics of patients having ulcers caused by *Haemophilus ducreyi* and TPE

The HD Class II analyzed in this study was more likely to cause longer-lasting ulcers than HD Class I (Table 5). Table 5 shows self-reported durations of limb ulcers caused by HD with respect to the molecular classification of HD classes. Class II caused longer-lasting ulcers compared to Class I, more frequently lasting more than six months (p < 0.001).

**Table 5:** Self-reported duration of limb ulcers caused by HD of Class I and II.

HD class	0–3 months	3–6 months	> 6 months
Class I	124 (94.7%)	7 (5.3%)	0
Class II	62 (82.7%)	6 (8%)	7 (9.3%)

The proportion of HD and TPE ulcers in patient age cohorts and the numbers of detected ulcers are shown in Table 6. While the proportion of HD infections among sampled individuals decreased gradually with increasing age (from 40.9% in the 0–9 years age group to 27.0% in the > 15 years age group), the decline of infections with TPE alone was more pronounced (from 47.7% - 17.1%, respectively). An even higher difference among age groups was found in patients with TPE and HD coinfection (Table 5, p < 0.001). While HD infection often leads to two or more ulcers, TPE infections less frequently cause multiple ulcers (Table 6, p = 0.026). Patients with TPE and HD coinfection, like TPE infections, often did not present with multiple ulcers.

**Table 6:** The proportion of HD and TPE ulcers in patient* age cohorts and the number of detected ulcers.

Age	HD positive patients (%)	TPE positive patients (%)	TPE + HD coinfection patients (%)
≤ 9 years	106 (40.9)	95 (47.7)	49 (62.8)
10–15 years	83 (32.0)	70 (35.2)	21 (26.9)
> 15 years	70 (27.0)	34 (17.1)	8 (10.3)
Total	259	199	78
Number of ulcers	HD positive patients (%)	TPE positive patients (%)	TPE + HD coinfection patients (%)
Single ulcer	167 (69.3)	149 (79.2)	57 (77.0)
Two or more ulcers	74 (30.7)	39 (20.8)	17 (23.0)
Total	241	188	74

*In our set of samples that were taken from children and young adults, slightly higher proportion of cases were found in females (53.4%) compared to males (46.6%); the difference was not statistically significant (therefore not included in Table 6).

## Discussion

HD causes cutaneous ulcers in children and sexually transmitted genital ulcers in adults. In this study, we analyzed swab samples from 1,081 ulcers diagnosed in the Yaws 3 Trial participants living in Namatanai, Papua New Guinea [4]. More than a third of samples (33.6%) were positive for HD DNA, which was more than the number of ulcer samples positive for the TPE DNA *polA* gene (28.5%; [10]), suggesting that HD is a slightly more common ulcer pathogen in the tested area than TPE. Of the 363 HD positive samples, a total of 72 samples (19.8%) were 16S rDNA positive but *dsrA* negative. Since there are six copies of 16S rRNA gene in the HD chromosome, qPCR detection of 16S rRNA gene could be more effective compared to single copy *dsrA* gene. In addition, some samples could contain HD with differences in the sequences of *dsrA* primer sites and could thus represent novel HD strain type(s). The 16S rRNA gene detection and *dsrA* typing were conducted at different time points which may have compromised the integrity of the DNA leading to potential failure in *dsrA* detection in *dsrA* negative samples.

In other studies, performed in the related geographical regions, including Vanuatu, Solomon Islands, and Papua New Guinea, HD PCR-positivity rates varied between 31.7% and 74% (54/73, 74%, [5]); (54/90, 60%, [8]); (13/41, 31.7%, [16]); (68/176, 38.6%, [6]) and our data are within this range. In four available studies from Africa performed in Ghana and Cameroon, the HD PCR-positivity rates varied more widely, between 9.2% and 84.6% (9/98 = 9.2%, [17]), (11/13 = 84.6%, [18]), (74/101, 73.3%, [19]), (82/271, 30.3%, [20]) suggesting that HD PCR-positivity rates may reflect other than geographical factors, possibly including population differences or additional epidemiological factors. In about 20% of HD patients, both HD and TPE DNA were detected in the same ulceration suggesting relatively common co-infection in many ulcers indicting similarities in epidemiological and clinical features between HD and TPE pathogens.

Even though TPE and HD infections were among the major causes of ulcer disease among child patients in Namatanai, PNG, almost half of the ulcers (43.4%) had an unknown etiology. It has been shown that other infectious agents, including *Streptococcus pyogenes* and anaerobic bacteria, are associated with this cutaneous ulcer syndrome in the Pacific region [21]. Additionally, staphylococcus [22], and Leishmania parasites have been shown to cause similar ulcers [5].

While genital ulcers in adults (chancroid) are more common in men than in women, in our cohort of children and young patients with cutaneous ulcers (i.e., less than a third of patients were older than 15 years), the positivity rate of HD infections was similar in both genders (53.4% in females and 46.6% in males). Consistent with our findings, a previous study conducted in Lihir, PNG [2], also reported similar rates in females (53.4%) and males (46.6%). However, several other studies showed a slight predominance of males among populations of similar age [5,16,18,19].

Altogether, eight different HD genotypes were found in this study, including four novel genotypes. All four novel genotypes belonged to Class I and were denoted as I.9, I.10, I.11, and I.12, in accordance with the previously introduced classification [7]. Similar genetic diversity of HD strains was found in a previous study from Lihir Island, PNG, where nine HD genotypes were identified [7]. However, unlike in the Lihir Island study, only a single Class II genotype was identified in our study i.e., II.3 while the II.1, II.4, and II.5 genotypes were not detected in our study. The I.3, I.4, and I.5 types were detected in both studies, while I.1 and I.8 genotypes were not identified in this study [7]. These findings suggest that different HD strains circulate in different infected populations. However, a more detailed information on circulating strains including genetic diversity in other chromosomal loci could be revealed by the whole genome sequencing either of HD DNA directly from patient’s samples or from cultivated HD isolates.

In general, the level of HD genetic diversity appears to be higher than that the observed diversity of TPE genotypes in the same set of samples, where only three different TPE genotypes were identified [10]. While these differences could reflect differences in the epidemiology and other characteristics of the two pathogens including infectivity rates, generation times and mutation rates, survival of pathogens in fomites, etc., the precise assessment of genetic variability would require whole genome sequencing of HD and TPE isolates. However, at least two conditions appear to contribute to this difference, i.e., the ability of HD to survive in the environment (which has not been described for TPE) and the very low mutation rate detected in TPE compared to other pathogens [23].

Even though HD and TPE pathogens share several similar characteristics, including obligate human pathogenicity, complex cultivation requirements with lower optimum temperatures (32–34°C), optimum growth under partially microaerophillic cultivation conditions, and induction of non-protective immunity after infections, we found that while the positivity rate of single HD infection is highest among the youngest children, it slowly decreased with age, on the other hand, single infections with TPE were seen to decrease with age more rapidly (an even higher rate of decrease was found in patients infected with both TPE and HD), indicating important differences in the epidemiology of the pathogens, likely reflecting differences in increasing degree of specific immunity to TPE and HD pathogens with increasing age. While some people appear resistant to HD infection due to innate immunity, there is no evidence of acquired immunity to HD either from natural or experimental infection [24]. At the same time, this finding suggests a higher proportion of non-TPE and non-HD ulcers in older children. Moreover, it has been found that TPE infections cause multiple ulcers less frequently than HD infections, suggesting differences in clinical presentations between the two infections.

The southern Matalai region was identified as the region with the lowest genotype diversity, with most infections caused by HD Class II, while HD Class I caused most infections in the central and northern part. Similar to this finding, a previous study [10] revealed that the TPE genotypes J_E_11 was detected in all areas of the Namatanai region. In contrast, additional minor genotypes, i.e., S_E_22 and T_E_13, were detected only in the central and north regions. These findings suggest important differences between southern Matalai, northen Sentral Niu Ailan, and the central Namatanai regions; it further indicates the existence of geographically-determined differences among common pathogen genotypes.

Analysis of patient characteristics revealed that HD Class II infections were more often associated with longer-duration ulcers than HD Class I infections or combined Class I and II infections (Table 4). Since the duration of limb ulcers caused by HD Class I and II was self-reported, we speculate that HD Class II may induce milder infections, prompting less medical (or other) treatment. This speculation is supported by the notable increase in the proportion of the I.10 strain type during Round 4 compared to Round 1 (Fig 4), which is consistent with the higher infectivity/pathogenicity of HD Class I and, more specifically, with the I.10 strain. Moreover, HD I.10 had the lowest average Ct values during the primary analysis of swab samples, suggesting a higher average number of bacteria in the ulcer than other HD types, including Class II.3 (Table 3). Another explanation of the over-representation of I.10 strains comprises asymptomatic colonization with I.10 [20] prior to azithromycin treatment and elimination of the infecting strains by antibiotics allowing I.10 to emerge later. Since it is estimated (based on whole genome comparisons) that both HD classes diverged about 1.95 million years ago, the genetic differences between HD of Class I and II could explain the observed difference in ulcer duration, reinfection of treated populations, and the number of bacteria present in ulcerations. It has been shown that Class II strains grow more slowly than Class I strains under the laboratory conditions, however, it is not clear whether this occurs also *in vivo* [25].

Another explanation of the notable increase in the proportion of I.10 strain types during Round 4 compared to Round 1 was the emergence of azithromycin resistance. Although there are no reports of mutations causing macrolide resistance in HD, there was a described clinical isolate that had minimal inhibitory concentration of 4 μg/ml for erythromycin [26,27]. In this report, we analyzed point mutations in the 23S rRNA genes of I.10 samples at baseline and post-antibiotic treatment in the DNA region known for macrolide resistance, including positions 2058 and 2059 which have been reported to confer the highest levels of resistance [28]. However, we did not find any consistently present mutations in the analyzed regions, suggesting that the I.10 strains still present in Round 4 remained susceptible to azithromycin. Since the lack of mutations could be a plausible explanation, there are additional alternative explanations. It is possible that macrolide resistance-encoding mutations in HD are located outside the analyzed region of the 23S rRNA genes and/or that mutations are present in other ribosomal components [29,30]. In addition, acquisition of genes encoding macrolide efflux pumps cannot be excluded as well. In two samples of the I.10 strain type from Round 4, we found two 23S rDNA point mutations, G1649A and C2092T (Table 4); however, the ability of these point mutations to confer macrolide resistance in HD remains unknown since the point mutations did not match any known mutant positions [28]. Although not all I.10 samples from Rounds 1 and 4 were analyzed due to ambiguous sequencing results in some samples, 80% of I.10 samples from both Rounds 1 and 4 (Table 4) were sequenced and evaluated. Since these mutations were only present in two out of 12 analyzed I.10 strains from Round 4, it is unlikely that these mutations are responsible for the observed azithromycin resistance and for the observed increase of I.10 strain types in Round 4.

Taken together, yaws eradication study in the Namatanai district of Papua New Guinea revealed that about one-third of all patients were infected with HD and that the prevalence of HD strains did not significantly decrease following mass azithromycin administration. Sequence typing of HD isolated from patients in the Namatanai region of Papua New Guinea, revealed multiple HD strains circulating in the area and greater genetic diversity of HD types compared to the yaws agent (i.e., TPE) present in the same area. Moreover, the infections caused by HD Class I and Class II strain types appear to differ in ulcer durations and infection resurgence after azithromycin treatment, suggesting important pathophysiological differences between HD Class I and HD Class II.

## Supporting information

S1 Material **Table A.** The data for the prevalence of HD genotypes from June 2018 to December 2019 as illustrated in Fig 1. **Table B.** Non-normalised dataset that was utilised to calculate the average Ct values for the dominant genotypes (I.3, I.5, I.9, I.10 and II.3) as presented in Table 3. **Table C.** Normalised dataset that was utilised to calculate the average Ct values for the dominant genotypes (I.3, I.5, I.9, I.10 and II.3) as presented in Table 3. **Table D.** Dataset for the genotypes detected before and after Azithromycin treatment as shown in Fig 4. **Table E.** The dataset used to generate the map showing the villages within the three local-level government areas where HD genotypes were detected as shown in Fig 3. **Table F.** The dataset used to create pie charts depicting the prevalence of strain types found in the three local-level government areas as shown in Fig 3.
(XLSX)
